# Supplementary material for: A Single-Celled Metasurface for Multipolarization Generation and Wavefront Manipulation
Source: Nanomaterials (Basel). 2022 Dec 6;12(23):4336. doi: 10.3390/nano12234336 (PMC9740456; doi:10.3390/nano12234336)
Supplement: Supplementary file 1 [file nanomaterials-12-04336-s001.zip › nanomaterials-2057241-supplementary.pdf]

Supplementary Materials

A Single-Celled Metasurface for Multipolarization Generation and Wavefront Manipulation

Ruonan Ji [1,*], Xin Guo [2], Zhichao Liu [3], Xianfeng Wu [1], Chuan Jin [3], Feng Liu [1], Xinru Zheng [1], Yang Sun [1] and Shaowei Wang [4,*]

[1] School of Physical Science and Technology, Northwestern Polytechnical University, Xi'an 710129, China
[2] Science and Technology on Low-Light-Level Night Version Laboratory, Xi'an 710065, China
[3] State Key Laboratory of Transient Optics and Photonics, Xi'an Institute of Optics and Precision Mechanics of CAS, Xi'an 710119, China
[4] State Key Laboratory for Infrared Physics, Shanghai Institute of Technical Physics, Chinese Academy of Sciences, Shanghai 200083, China
* Correspondence: jiruonan@nwpu.edu.cn (R.J.); wangshw@mail.sitp.ac.cn (S.W.)

Simulated spin-flipped component reflectance color maps of the unit cells

The reflectance of the spin-flipped components was simulated; the structural parameters were chosen as p=700 nm, r=120 nm, w=80 nm, t_1=200 nm, t_2=180 nm, t_3=100 nm, and β=60º; and the results are shown in Figure S1. The metallic material in the MIM structure was chosen as aluminum and gold, respectively. Obviously, the values of R_{LL} and R_{RR} can be both improved by replacing aluminum with gold with lower loss in this range. The average values of R_{LL} and R_{RR} in the wavelength range of 1.10-1.90 μm can both be above 0.8. Moreover, the reflectance can be further improved by optimizing the structure parameters.

In this paper, the meta-atom was designed as a MIM structure due to the cavity enhancement effect on the reflectance. In order to verify the performance, the reflectance color maps of MIM and monolayer structure were simulated and compared. It can be found that the spin-flipped components' reflectance values of the monolayer meta-atom are quite limited; the maximum value was lower than 0.15. Thus, the design of a MIM structure indeed obviously enhances the efficiency of the metasurface.

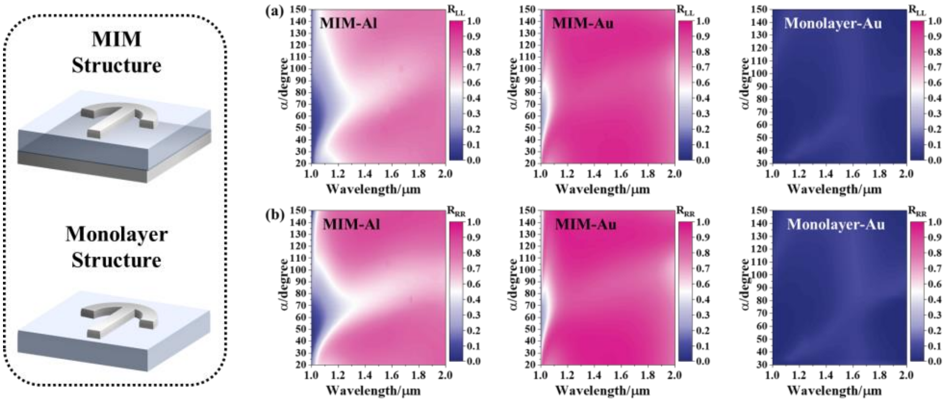

Figure S1. Simulated reflectance color maps of MIM and monolayer structure with metallic material of aluminum (a) R_{LL} and (b) R_{RR} and gold. Here, β is fixed at 60° while α varies from 20° to 150°. The permittivity of aluminum and gold were taken from Ref. [1].

References

1. Palik, E. D., HANDBOOK OF OPTICAL-CONSTANTS. Journal of the Optical Society of America A-Optics Image Science and Vision 1984, 1 (12), 1297-1297.
